# Detection of Yeast-like Symbionts in Brown Planthopper Reared on Different Resistant Rice Varieties Combining DGGE and Absolute Quantitative Real-Time PCR

**DOI:** 10.3390/insects13010085

**Published:** 2022-01-13

**Authors:** Chengling Lai, Yun Hou, Peiying Hao, Kun Pang, Xiaoping Yu

**Affiliations:** Zhejiang Provincial Key Laboratory of Biometrology and Inspection & Quarantine, College of Life Sciences, China Jiliang University, Hangzhou 310018, China; lchenglai@163.com (C.L.); lyn199709@sina.com (Y.H.); haopeiy@163.com (P.H.)

**Keywords:** brown planthopper, yeast-like symbionts, resistant rice, DGGE, quantitative real-time PCR

## Abstract

**Simple Summary:**

The brown planthopper (BPH) is an important pest that causes huge losses in rice production. The promotion and use of insect-resistant rice varieties is an important way to control BPH. However, in practice, BPH can adapt to resistant rice within several generations. Endosymbionts may be one of the reasons for the rapid adaptation of BPH to resistant rice. The BPH harbor yeast-like symbionts (YLS) in their abdomen, and YLS are essential for the nutrition, development, and reproduction of BPH. Our previous report showed that among the YLS communities detected in BPH, *Ascomycetes* symbionts, *Pichia*-like symbionts, and *Candida*-like symbionts were the three dominant populations of YLS. In this study, PCR-DGGE and absolute quantitative real-time PCR were used to detect the variations of three dominant YLS in BPH across different nymph ages and on different resistant rice varieties. The results showed that the total number of YLS gradually increased from the first instar to adulthood, but decreased in the fifth instar nymph, when BPH were reared on the susceptible rice variety TN1. The rice-resistant varieties, Mudgo, ASD7, and RH have more significant inhibitory effects on the three dominant YLS in the first and second generations of BPH. However, the numbers of the three dominant YLS were all recovered from the third generation of BPH. *Ascomycetes* symbionts were the most dominant strain among the three YLS.

**Abstract:**

The brown planthopper (BPH), *Nilaparvata lugens*, is a serious pest of rice throughout Asia. Yeast-like symbionts (YLS) are endosymbionts closely linked with the development of BPH and the adapted mechanism of BPH virulence to resistant plants. In this study, we used semi-quantitative DGGE and absolute quantitative real-time PCR (qPCR) to quantify the number of the three YLS strains (*Ascomycetes* symbionts, *Pichia*-like symbionts, and *Candida*-like symbionts) that typically infect BPH in the nymphal stages and in newly emerged female adults. The quantities of each of the three YLS assessed increased in tandem with the developing nymphal instar stages, peaking at the fourth instar stage, and then declined significantly at the fifth instar stage. However, the amount of YLS present recovered sharply within the emerging adult females. Additionally, we estimated the quantities of YLS for up to eight generations after their inoculation onto resistant cultivars (Mudgo, ASD7, and RH) to reassociate the dynamics of YLS with the fitness of BPH. The minimum number of each YLS was detected in the second generation and gradually increased from the third generation with regard to resistant rice varieties. In addition, the *Ascomycetes* symbionts of YLS were found to be the most abundant of the three YLS strains tested for all of the development stages of BPH.

## 1. Introduction

Rice (*Oryza sativa*) is the main cereal crop for more than 50% of the world’s population and is known to provide energy and nutrition. However, it suffers from a total of around 800 pests in the field and storage stages [1]. The brown planthopper (BPH), *Nilaparvata lugens* (Stål) is a rice pest that sucks sap from phloem tissues of the rice stem [2,3]. The main hazards of BPH to rice are demonstrated in three aspects, namely, in direct ingestion, oviposition, and in disseminating or inducing rice diseases. In general, when BPH breaks out in large numbers, it causes immeasurable losses to rice production [4].

The use of chemical methods to control rice BPH will result in many serious problems, including toxicity to the natural enemies of BPH, an increase in the total production cost of rice, and the possible serious damage to the ecosystem and human health [5,6]. Therefore, it is widely believed that the selection and breeding of resistant rice varieties is an environmentally friendly strategy important for suppressing BPH populations at a lower economic cost [7,8,9,10]. However, the wide and successive deployment of resistant rice varieties has resulted in a new virulent BPH population with an ability to adapt to the resistance of rice varieties [11,12]. As of yet, the precise nature of the resistance-adapting mechanism in BPH is unknown. A better understanding of the mechanism of interaction between BPH virulence and rice resistance may significantly enhance the breeding of resistant rice varieties and the deployment of rice varieties in controlling BPH populations.

Recently, more attention has been focused on the relationship between insects and the microbes they harbor. Symbionts have been found to contribute to the nutrition, development, reproduction, speciation, and defense against natural enemies of their host insect [13,14,15,16]. Endosymbiont bacteria and yeast-like endosymbionts (YLS) play an important role in BPH growth, reproduction, and nutrition. The former is mainly composed of *Wolbachia* and *Arsenophonus*, and the results showed that the two bacteria mainly existed in fat bodies and were transmitted through maternal lines [17]. *Wolbachia* can provide biotin and riboflavin to BPH to enhance its reproductive ability [18]. Relevant studies have found that *Wolbachia* in BPH also has cytoplasmic incompatibility, which can help us to control the transmission of rice viruses and inhibit the population of BPH [19,20]. Hu et al. studied the number of BPH symbiont bacteria on TN1, Mudgo, and asd7 rice, and detected changes in the bacterial number and structure during their adaptation to different resistant rice by fluorescent PCR. The results showed that *Chryseobacterium* was the dominant bacterium of the BPH population, but had no significant effect on the virulence variation of BPH, although *Serratia* and *Arsenophonus* may affect the virulence variation of BPH [21]. The latter mainly includes *Ascomycetes* symbionts, *Pichia*-like symbionts, and *Candida*-like symbionts, which exist in the abdominal fat, and are transmitted to the progeny via the ovarian vertical [22,23,24]. YLS can provide BPH with rare nutrients that are lacking in plant phloem amino acids [25]. Studies have found that BPH nymphs that do not contain YLS experience weight loss and slower growth compared to normal BPH nymphs [26]. Related studies have shown that YLS plays a role in amino acid metabolism through the recycling of uric acid [25]. Lu et al. found that, in different resistant rice, the development duration of BPH nymphs was significantly prolonged, the survival rate was significantly decreased, and the number and enzymatic activity of YLS in the nymphs were reduced [26]. In the following second and third generations, the nymph performance and the number of YLS gradually recovered. This indicates that YLS may be related to the variation of BPH virulence and its adaptation to rice resistance. Therefore, one hypothesis is that BPH endosymbionts play an important role in the resistance adaptation of BPH.

BPH has repeatedly demonstrated the ability to adapt to resistant rice varieties, within a few generations, after continuous feeding on specific resistant rice varieties in the laboratory or on the paddy field. These findings raise the following questions: What is the relationship between YLS and the adaptation of BPH to resistant rice varieties? How does the number of YLS vary with the developmental stage of brown planthopper in rice varieties? However, YLS are endosymbionts found in the BPH body and are difficult to culture in vitro, most probably because of their unique environmental requirements, which are specifically provided by the insect host. Identifying and understanding the relevance of YLS and its insect host, based on its morphological and physiological characteristics, is considered a major challenge.

In recent years, with the development of molecular biology technology, we have been able to use emergent technologies to identify YLS cultured in vitro, as well as undiscovered or unidentified YLS. Some studies used the PCR amplification of rDNA or ITS (internal transcribed spacer) sequences and evaluated the complex microbial population using denaturing gradient gel electrophoresis (DGGE) technology [27,28,29,30]. Our previous research results showed that, compared with studies using traditional culture techniques or based on 18S rDNA sequences, nested PCR-DGGE provides a more comprehensive estimate of the diversity of microbial communities in BPH [31]. Using the PCR-DGGE strategy, both identified and unidentified or unreported fungi were reported. Moreover, YLS such as *Ascomycetes* symbionts, *Pichia*-like symbionts, and *Candida*-like symbionts were found to be dominant among the detected YLS communities in BPH [23,32,33]. In this study, PCR-DGGE and absolute quantitative real-time PCR were used to quantitatively determine the variation of three dominant YLS in BPH in different nymph instars and different resistant rice varieties. The data presented here provide a basis for exploring the relationship among the YLS number or species, BPH instar, and resistant rice varieties, and further elucidate the resistance-adapting mechanisms of BPH-YLS symbionts to resistant rice varieties.

## 2. Materials and Methods

### 2.1. Rice Varieties

The rice varieties used in this study included susceptible variety TN1 (no resistant gene) and the following three resistant rice varieties: Mudgo (carrying Bph1 gene for resistance), ASD7 (carrying Bph2 gene for resistance), and Rathu Heenati (RH) (carrying Bph3 gene for resistance) [11].

### 2.2. Collection and Culture of Insects

Adult BPH were obtained from rice fields in Hangzhou, China (120°12′ E, 30°16′ N). The BPH populations were raised on rice variety TN1 and placed in an artificial climate room with an environmental condition of 26.0 ± 1.0 °C a humidity of 70.0–80.0%, and light: darkness = 16 h:8 h.

To assess the relationship between the dominant YLS and different nymphal instars, 500, 400, 300, 200 BPH from the 1st–2nd, 3rd, 4th, 5th instars, and 100 newly emerged female adults were collected for further YLS isolation. In order to examine the relationship between the dominant YLS and different resistant rice varieties, we placed 100 gravid female adults from the susceptible rice variety TN1 onto the resistant rice varieties of Mudgo, ASD7, and RH, for consecutive rearing across more than 8 generations. The 100 newly emerged female adults were collected from the 1st to the 8th generation on each of the three resistant rice varieties, and on the susceptible rice variety TN1 as the control.

### 2.3. Isolation of YLS from BPH

The YLS were extracted from newly emerged female adult BPH fat body and the entire instar BPH body, and the methods for purification used by Noda and Omura were slightly modified [34]. The following method was used: completely immerse the worm body in 75% ethanol for 3 min, then dissect the fat body from the sterilized BPH, and break it in 0.02 M phosphate-buffered saline (PBS) with pH 7.4 Percoll (Pharmacia, NJ, USA) to obtain a homogeneous solution. Next, add the PBS solution to the homogenate to a final concentration of 30%, and centrifuge at 2000× *g* for 10 min. Discard the supernatant, add 250 mM sucrose and 75% Percoll (Pharmacia, NJ, USA) in PBS to the pellet, and centrifuge at 10,000× *g* for 20 min. The YLS were concentrated in the 65–85% area of the Percoll gradient, so that YLS were collected from this area and the total number was counted with a hemocytometer under a microscope (Leica, Wetzlar, Germany) and was then calculated according to the formula simulated by Chen et al. [11].

### 2.4. Total DNA Extraction

Genomic DNA from YLS was extracted with a Yeast DNA Mini Kit (Tiangen Biotech Co Ltd., Beijing, China), which is a spin-column purification method based on the enzymolysis of yeast cells with recombinant lyticase enzymes (Sigma, St. Louis, MO, USA).

### 2.5. PCR Amplification

The extracted DNA samples were analyzed using PCR-DGGE fingerprinting technology. Our previous work revealed the existence of three dominant YLS (*Ascomycetes* symbionts, *Pichia*-like symbionts and *Candida*-like symbionts) in the fat body of BPH; therefore, the primers were designed according to the obtained Internally Transcribed Spacer (ITS) region sequence of these YLS, respectively (Table 1). The primers were synthesized by Shanghai Sunshine Biotechnology Co., Ltd. Furthermore, 310F and 310R were designed to amplify the ITS fragment of the *Ascomycetes* symbionts, while 305F and 305R were designed to amplify the ITS fragment of the *Pichia*-like symbionts and 248F and 248R were designed to amplify the ITS fragment of the *Candida*-like symbionts. In order to improve the stability of DNA fragments during electrophoresis, a GC clip was added to the 5′ end of 310F, 305F and 248F. Amplification was performed in the 50 μL volume that contained approximately 30 ng DNA, 10 × Ex buffer 5 μL, dNTP 0.8 mM, primers 0.5 mM each, Ex Taq polymerase 1.25 U (TaKaRa, Kyoto, Japan,). The touchdown PCR were cycled in a thermal cycler, for which the specific procedure is shown in Table 2. Then, the band was cut from the gel and the DNA was purified with the DNA Purification Kit (QIAGEN, Dusseldorf, Germany), according to the instructions provided.

### 2.6. Denaturing Gradient Gel Electrophoresis

The variations of three dominant YLS were measured by semi-quantitative DGGE. DGGE was performed with the Bio-Rad Protean 11 system. The electrophoresis conditions were 8% polyacrylamide (acrylamide: bisacrylamide = 37.5:1); the denaturation range was 20–50%, 10 μL of the PCR product, with the addition of a 2 μL 6 × loading buffer which was mixed and loaded, 80 V, 12.5 h. After electrophoresis, a routine silver staining protocol was used for the detection of DNA in the DGGE gel [35]. The silver dyeing conditions were as follows: (1) rinse twice with an appropriate amount of ultrapure water, for 1 min each time; (2) discard the ultrapure water, add an appropriate amount of dyeing solution (0.2% silver nitrate, 10% absolute ethanol, 0.5% glacial acetic acid), shake slowly for 15 min; (3) discard the staining solution, quickly rinse the gel with ultrapure water, then pour the pre-cooled developer solution (37% formaldehyde, 3% sodium hydroxide), and shake until the band is clearly distinguished; (4) discard the developing solution, quickly rinse the gel with ultrapure water, add an appropriate amount of stop solution (10% absolute ethanol, 0.5% glacial acetic acid), shake for 2 min, discard the stop solution, rinse with ultrapure water several times and scan and record the results using the Bio-Rad gel imaging analyzer (Ann Arbor, MI, USA).

### 2.7. Absolute Quantitative Real-Time PCR Analysis

The variations of three dominant YLS in BPH fat body were also measured by qPCR. The qPCR analysis performed on the IQTM5 multicolor real-time PCR detection system (Bio-Rad, CA, USA) and the absolute quantification of the three YLS using the pMD18-TTM cloning kit (TaKaRa, Kyoto, Japan) were all borrowed from the method of Cao et al. and were also specific to the results of the dissolution curve analysis that was carried out [36,37,38].

### 2.8. Data Analysis

SPSS version 16.0 was used for a more detailed statistical analysis, such as the analysis of variance (ANOVA). Mean comparisons were conducted using Duncan’s multiple range test.

## 3. Results

### 3.1. The Variation and the Number of the Dominant YLS in Different BPH Instars

**Figure 1:**
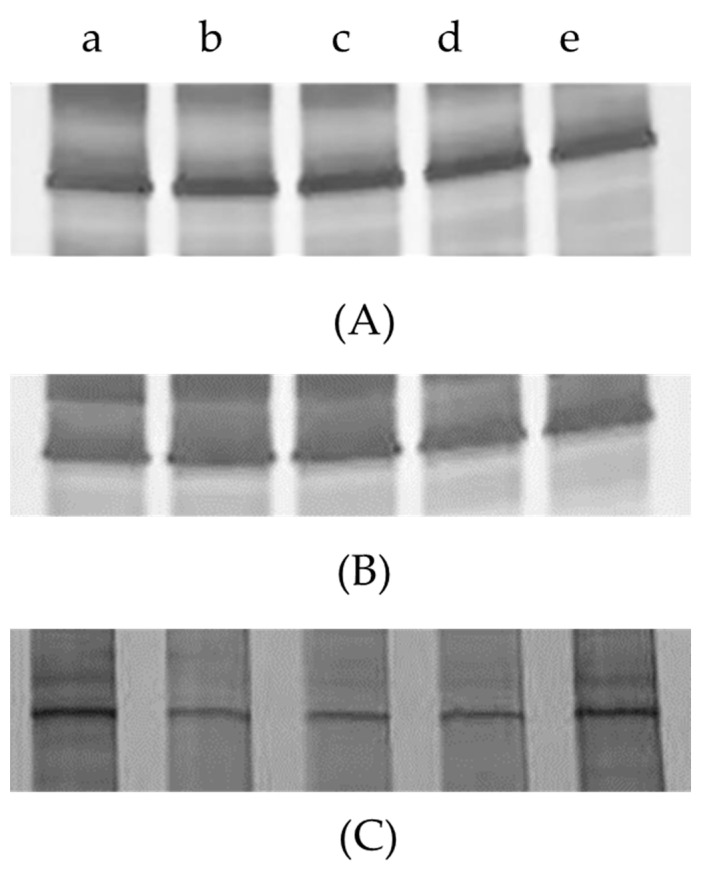
The DGGE analysis of variations of the three dominant YLS *Ascomycetes* symbionts (**A**), *Pichia*-like symbionts (**B**), *Candida*-like symbionts (**C**) in different nymphal stages. a: 1st and 2nd instar; b: 3rd instar; c: 4th instar; d: 5th instar; e: newly emerged female adult.

Using the DGGE analysis of three dominant YLS in different BPH instars, distinct ITS banding patterns were observed, and each sample profile displayed a unique banding pattern (Figure 1). In each case of three dominant YLS (*Ascomycetes* symbionts, *Pichia*-like symbionts and *Candida*-like symbionts), no significant quantitative difference in band intensity was found among the different developmental stages of BPH, while the intensity of bands representing *Ascomycetes* symbionts and *Pichia*-like symbionts was much stronger than that of bands representing *Candida*-like symbionts at the same BPH stages. The above results suggest that the number of *Ascomycetes* symbionts and *Pichia*-like symbionts was higher than that of *Candida*-like symbionts in BPH bodies.

**Figure 2:**
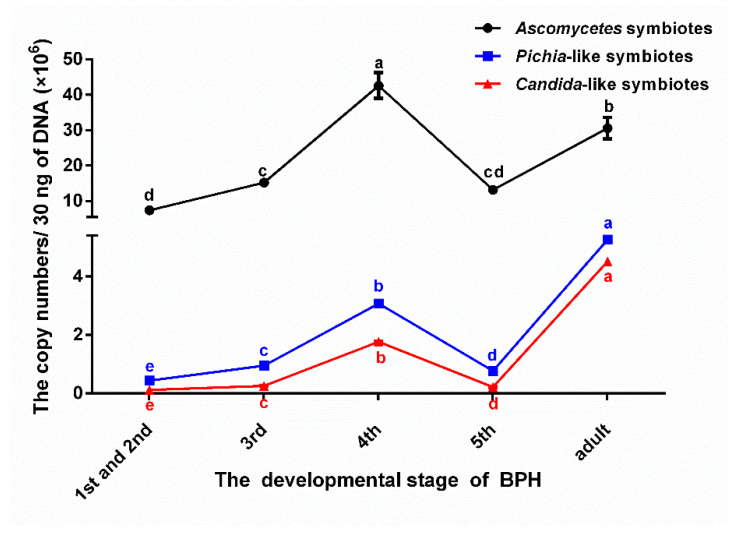
The copy number (×10^6^) of three dominant YLS of BPH in the different developmental stages. The 1st and 2nd, 3rd, 4th, 5th represent the instar nymph stage; adult represents newly emerged female adults. There were significant differences between different lowercase letters (*p* ≤ 0.01, one-factor ANOVA and Tukey’s multiple comparisons).

As shown in Figure 2, among the three dominant YLS (*Ascomycetes* symbionts, *Pichia*-like symbionts, and *Candida*-like symbionts) of BPH, the copy numbers of *Ascomycetes* symbionts were much higher than those of the other two strains during any of the nymph instar. The data indicate that the *Ascomycetes* symbionts, as YLS, are most prolific in the BPH fat body. These results are consistent with an aforementioned phenomenon detected using DGGE, and the *Ascomycetes* symbionts, as the first endosymbionts found in BPH, were confirmed as the dominant strain in YLS [39]. In addition, under the different nymphal instars of BPH, the variation of the three YLS was found to have a similar trend, i.e., the number of the three YLS increased with the developing nymphal instar stages, peaking at the fourth instar stage and declining significantly at the fifth instar stage. However, the quantities of YLS recovered sharply within the emerging adult females.

### 3.2. The Variation and the Number of Dominant YLS in Different BPH Generations Rearing on Resistant Rice Varieties

**Figure 3:**
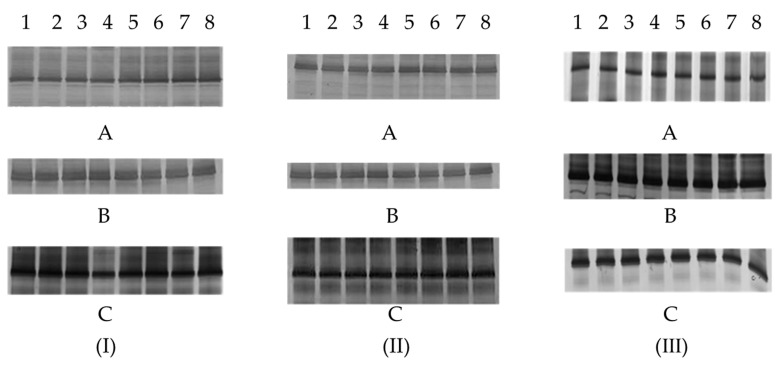
The DGGE analysis of variations of three dominant YLS *Ascomycetes* symbionts (**I**), *Pichia*-like symbionts (**II**), *Candida*-like symbionts (**III**) of BPH rearing on three different resistant rice varieties Mudgo (**A**), ASD7 (**B**), and RH (**C**), respectively. Numbers 1–8 represent the 1st generation to the 8th generation of BPH female adult.

DGGE fingerprinting technology was also used to analyze the variation of the three YLS of BPH populations reared on the rice varieties of Mudgo, ASD7 and RH (Figure 3). In the case of BPH rearing on the Mudgo rice variety, the intensity of the *Ascomycetes* symbionts band was fainter than that of the BPH rearing on two other rice varieties, which led to the assumption that the *Ascomycetes* symbionts are much more sensitive to the Mudgo rice variety than other two YLS strains (Figure 3Ⅰ). Regardless of the rice variety, the copy number of *Candida*-like symbionts did not change to a meaningful degree according to the intensity of the band (Figure 3Ⅲ). Therefore, we used qPCR to characterize the copy number of all three YLS in detail.

**Figure 4:**
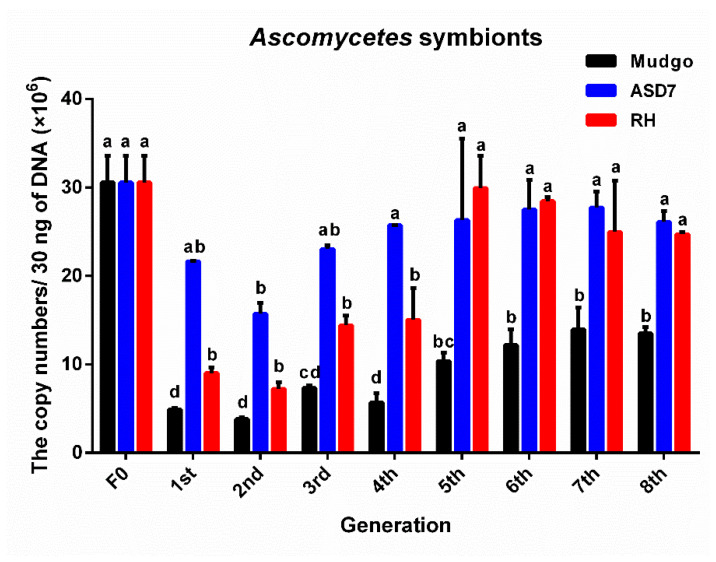
The copy number (×10^6^) of *Ascomycetes* symbionts of BPH cultivated on three BPH-resistant rice varieties. The F0 generation is BPH raised on TN1 rice. There were significant differences between different lowercase letters (*p* ≤ 0.01, one-factor ANOVA, and Tukey’s multiple comparisons).

**Figure 5:**
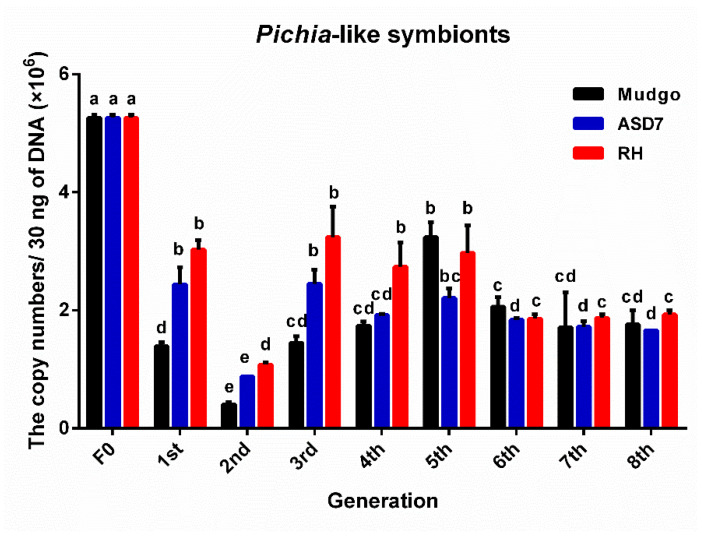
The copy number (×10^6^) of Pichia-like symbionts of BPH cultivated on three BPH-resistant rice varieties. The F0 generation is BPH raised on TN1 rice. There were significant differences between different lowercase letters (*p* ≤ 0.01, one-factor ANOVA, and Tukey’s multiple comparisons).

**Figure 6:**
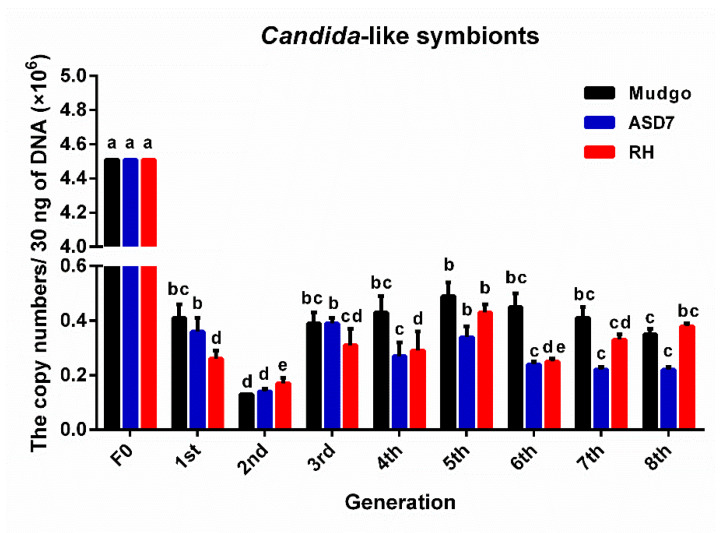
The copy number (×10^6^) of *Candida*-like symbionts of BPH cultivated on three BPH-resistant rice varieties. The F0 generation is BPH raised on TN1 rice. There were significant differences between different lowercase letters (*p* ≤ 0.01, one-factor ANOVA, and Tukey’s multiple comparisons).

As shown in Figure 4, the copy number of *Ascomycetes* symbionts in the Mudgo rice variety varied most obviously as compared to those on the other two resistant rice varieties (F = 82.687, df = 8, 18, 26, *p* ≤ 0.01), indicating that the greatest changes in abundance of *Ascomycetes* symbionts occurred on the Mudgo variety. As show in Figure 5 and Figure 6, on all tested resistant rice varieties, the copy number of *Pichia*-like symbionts and *Candida*-like symbionts decreased to a similar level, and the number of these two symbionts began to decline again in the sixth generation. According to the data summarizing the comparison of each dominant YLS on different rice varieties, which are presented in Figure 4, Figure 5 and Figure 6, the number of each dominant YLS reached the minimum level in the second generation, and increased gradually from the third generation. It is worth mentioning that there was a much higher number of *Ascomycetes* symbionts found on all three rice varieties as compared to the other two YLS, which is consistent with the above-mentioned data for different nymphal periods.

## 4. Discussion

Over the past 40 years, the cultivation and use of resistant rice varieties has become the primary strategy for BPH control. Therefore, planting resistant rice is widely regarded as the most effective way to avoid or reduce the production loss caused by BPH. BPH contains yeast-like symbionts (YLS) in its abdominal fat body, and the YLS in BPH grow in tandem with the host and exist in every stage of the development of the BPH. Some indications showed that YLS may be involved in various metabolic pathways, and may play a role in the growth and development of its host [40,41]. For this reason, it is of interest to survey the variations of dominant YLS in each nymphal period of BPH. Furthermore, the YLS was thought to be relevant to the adaptation of BPH to resistant rice varieties, since YLS are found to be closely linked with BPH performance on new rice varieties. Understanding the characteristics and variations of dominant YLS in BPH on different resistant rice varieties is essential for comprehensively studying the adaptation mechanism of BPH to resistant rice varieties.

Firstly, semi-quantitative DGGE technology and absolute qPCR were employed to assess the number of the three dominant YLS in the different BPH developmental stages. The results demonstrated that the number of *Ascomycetes* symbionts, *Pichia*-like symbionts, and *Candida*-like symbionts increased with the development of BPH, but decreased in the fifth instar. This is slightly different from the results reported by Lu et al. who found that the number of YLS increased from the first instar to adults, which may be due to different observation and detection techniques [26]. In addition, the number of dominant YLS decreased significantly when the BPH developed to the fifth instar. This may be because the fifth instar of BPH is the transitional stage from nymph to adult, which means that the regulation mechanism of YLS may be more prominent this stage. In our experiment, YLS were obtained from the fat body of adults, while nymph-extracted YLS were obtained from the whole insect. It is worth mentioning that YLS exists in the hemolymph and ovariole [23]. The amount of free YLS in the hemolymph is still unknown, and presents a potential research avenue for further study.

## 5. Conclusions

Our results showed that resistant rice varieties, such as Mudgo, ASD7, and RH all remain resistant to YLS in the first and second generations of BPH. However, starting from the third generation of BPH, the numbers of three dominant YLS were recovered. It seems that a reduction in YLS is associated with the development and metabolism of their host BPH, mostly considered to be a response of the BPH and YLS complex to an adverse and resistant environment, resulting in a decrease in dominant YLS in the first two generations after the transference of BPH from the susceptible variety to a resistant variety. After the third generation, the BPH gradually adapted to resistant plants as the number of YLS increased. Similarly, in previous studies on *Bemisia tabaci*, it was found that the number of symbionts in *Bemisia tabaci* populations from different plants fluctuated significantly before the third or fourth generation, after inoculation on different plants, and there was no significant change after that [42]. It can be seen that there seems to be a relationship between the variation of YLS and resistant rice varieties. Previous studies on aphid symbionts have shown that the types and quantities of symbionts carried by aphids are related to the ecological environment of the host plants, and aphids in the same ecological environment all over the world have obtained the same or closely related symbiont genotypes [43]. Moreover, there is evidence that endosymbionts can significantly improve the adaptability of insects to specific host plants [44,45,46]. Combined with the response of other insect symbionts to plant stress, we propose that the function of YLS in BPH is closely linked with the adaptation of BPH to resistant rice varieties. Therefore, our future research will explore the role of YLS in the growth and development of BPH, and further examine its potential role in the adaptation of BPH to resistant rice varieties.

## Figures and Tables

**Table 1:** Primers used for PCR or absolute quantitative real-time PCR in this study.

Primer	Sequence (5′→3′)
310FGC *	TCCCTCTGTGGAACCCCAT
310R	GGCGGTCCTAGAAACCAACA
305FGC *	GCGAGTACTGGACCCAAC
305R	GGCCTGCTTTGAACACTCT
248FGC *	CTGCGGACGGATCATTACA
248R	GCCAAACCAAAGCAAGAGTTC
q310F	TCCCTCTGTGGAACCCCAT
q310R	GGCGGTCCTAGAAACCAACA
q305F	GCGAGTACTGGACCCAAC
q305R	GGCCTGCTTTGAACACTCT
q248F	CTGCGGACGGATCATTACA
q248R	GCCAAACCAAAGCAAGAGTTC

* GC clamp was added to the 5′ end of 310F, 305F, 248F. GC-clamp: CGCCCGCCGCGCCCGCGCCAGCCGCCGCGCCCGCCGCG.

**Table 2:** The touchdown PCR cycle procedure in this study.

	Temperature	Time	Number of Cycles
Pre denaturation	94 °C	5 min	1×
Denaturation	94 °C	45 s	20 × (annealing: 2 cycles per temperature)
Annealing	57–67 °C	45 s	
Extension	72 °C	1 min	
Annealing	58 °C	45 s	20×
Extension	72 °C	10 min	1×

## Data Availability

All data are included in figures, or can be obtained by contacting the corresponding author.
